# Sleep–wake changes and incident depressive symptoms in midlife women

**DOI:** 10.1038/s41598-024-66145-3

**Published:** 2024-07-02

**Authors:** Jing Luo, Song Lin

**Affiliations:** 1 School of Rehabilitation, Jiangsu College of Nursing, Huaian, 223003 Jiangsu China; 2 Department of Clinical Nutrition, The Affiliated Huaian No.1 People’s Hospital of Nanjing Medical University, Huaian, 223300 Jiangsu China

**Keywords:** Depressive symptoms, Depression, Sleep, Circadian rhythms, Women, Midlife, Prospective cohort, Psychology, Risk factors, Disease prevention, Public health

## Abstract

Our study aimed to investigate the relationship between sleep–wake changes and depressive symptoms events among midlife women. We enrolled 1579 women aged 44–56 years who had no clinically relevant depressive symptoms at baseline. Depressive symptoms were assessed at each visit using the Center for Epidemiologic Studies Depression scale. At the third and fourth follow-up visits, women reported their sleep habits. The sleep midpoint was defined as the time to fall asleep plus one-half of the sleep duration. Sleep–wake changes were determined by the difference in the midpoint of sleep between the third and fourth visits, which were 1 year apart. The median follow-up time was 7 years (range 1–7 years). Cox proportional hazard models were fitted to calculate hazard ratios and 95% confidence intervals for the incidence of depressive symptoms associated with sleep–wake changes. After adjusting for potential confounding factors, the hazard ratio (95% confidence interval) of depressive symptoms for severe sleep midpoint changes was 1.51 (1.12, 2.05) compared with mild sleep midpoint changes. This relationship remained statistically significant and changed little when additionally controlling for sleep duration, sleep quality, insomnia symptoms, use of sleep medications, use of nervous medications, glucose, insulin, lipids, dietary energy intake, and C-reactive protein. Our findings indicate that exposure to long-term severe sleep–wake changes increases the risk of depressive symptoms in midlife women.

## Introduction

Sleep–wake changes has been implicated in the progression of metabolic diseases^1,2^, cardiovascular diseases^3,4^, neurodegeneration^5,6^, and psychiatric disorders^7–9^. Therefore, for individuals, maintaining a certain level of sleep–wake rhythm homeostasis is essential for wellness, especially for mental health.

Epidemiological studies have suggested a potential relationship between sleep–wake changes and depression. According to a study by Kuula et al., there is a strong association between deviations in circadian rhythms and psychiatric problems, such as depression^10^. Participants with irregular and permanent night shifts are associated with increased risk of incident depression^11^. Dashti et al. reported that later sleep midpoint was associated with a higher risk of major depressive disorder after adjustment for age, gender, race/ethnicity, employment status, body mass index (BMI), smoking status, and education attainment in Mass General Brigham Biobank cohort study^12^. Delving deeper through a Mendelian randomization meta-analysis, genetically proxied earlier diurnal preference is associated with a lower risk of depression^13^. However, these results are mainly based on a single assessment of sleep schedules, so the impact of long-term changes in sleep–wake rhythms on the onset of depression cannot be explored in depth.

The menopausal transition is associated with increased risk of sleep disturbances^14^. The relative amplitude and stability of circadian rhythms are lower in perimenopausal women than in premenopausal women^15^. Additionally, women at this stage are more likely to develop depressive symptoms^16^. These phenomena mentioned above may be related to the fluctuation of ovarian hormones during the menopausal transition. However, studies on sleep–wake rhythms and depression have rarely focused on menopausal status and hormone levels. Therefore, we aim to explore the potential relationship between sleep–wake changes and the incidence of depression in women during the menopausal transition. Due to the complexity and time-consuming nature of directly monitoring the entire dynamic changes of the sleep–wake changes, researchers typically rely on a series of proxy measures to assess this cycle. Among these, the sleep midpoint is particularly selected as a key indicator for research. On one hand, the sleep midpoint is closely related to an individual’s circadian rhythm, reflecting the shift of the biological clock. By analyzing changes in the sleep midpoint, we can gain insights into the stability of an individual’s biological clock and its synchronization with the environmental light–dark cycle. On the other hand, compared to the times of falling asleep and waking up, the sleep midpoint is less susceptible to random factors, demonstrating better stability and repeatability, making the monitoring of this indicator crucial for understanding the impact of the sleep–wake changes on health. Thus, we hypothesize that sleep–wake changes (the change in the midpoint of sleep over 2 years) is related to the depression risk in women during the menopausal transition, while the association may be affected by the menstrual state and sex hormones.

## Methods

### Study population

Data were collected as part of the SWAN, a multi-site, multi-racial/ethnic, longitudinal community-based study of the US midlife women. Enrollment began in January 1996, and a total of 3302 eligible premenopausal women (aged 42 to 52 years) completed their initial visit at 7 clinical sites (Boston, MA; Chicago, IL; Detroit, MI; Los Angeles, CA; Newark, NJ; Oakland, CA; and Pittsburgh, PA) in December 1997. Eligibility criteria were menstruating, had a uterus and at least one intact ovary, and not using hormone medications in the past 3 months. The SWAN cohort was followed almost annually during the menopausal transition. The project was approved by the institutional review boards at all sites, and informed consent was provided by all participants at each visit. This study was conducted in accordance with the Strengthening the Reporting of Observational Studies in Epidemiology (STROBE) guidelines. More details on study design have been published previously^17^.

To assess changes in sleep–wake rhythm, women who participated in the sleep habits survey at two consecutive visits were included. Therefore, the present analysis includes eligible women who completed in the sleep survey at both the third (treated as the baseline for the current analysis, 1999–2001, n = 2709) and forth (2000–2002) visits. We excluded 366 women who did not have sufficient data to calculate changes in the midpoint of sleep and 460 women with prevalence depressive symptoms [Center for Epidemiologic Studies Depression (CES-D) Scale ≥ 16] at baseline. We further excluded 304 women who underwent bilateral salpingo-oophorectomy, hysterectomy, or hormone-replacement therapy at baseline, leaving 1579 women entered the risk set and followed from the third visit (1999–2001) through the tenth visit (2006–2008).

### Sleep

At the third and fourth follow-up visits, women were requested to provide an accurate description of their sleep habits [bed time, get up time, hours of sleep per night and sleep quality (very good, good, bad, and very bad)] on most nights during the past month through a structured questionnaire. The sleep midpoint was defined as the time to fall asleep plus one-half of the sleep duration. The difference in the midpoint of sleep between the third and fourth visits, spaced 1 year apart, was calculated and then categorized into three groups: mild-change (< 1 h), moderate-change (1 to 2 h), and severe-change (> 2 h).

At baseline of our study, women reported the frequency of sleep problems over the previous 2 weeks (trouble falling asleep, waking up several times per night, and waking up earlier than planned and unable to fall asleep again). Each of these responses was dichotomized as “no/infrequent” (≤ 2 times per week) or “frequent” (≥ 3 times per week). Insomnia symptoms were defined as any of the three symptoms being reported as “frequent”.

### Depressive symptoms

Depressive symptoms were assessed using a 20-item CES-D scale at each follow-up visit. The response categories for each item “rarely or none of the time”, “some or a little of the time”, “occasionally or a moderate amount of the time”, and “most or all of the time” were given a score ranging from 0 to 3. As a severity measure, CES-D total score was calculated as the sum of the points in each item ranging from 0 to 60. A meta-analysis showed that CES-D score ≥ 16 had sensitivity of 87% and specificity of 70% for standardized diagnoses of major depression in adults^18^. Therefore, in the present study, CES-D score of 16 or higher was used to identify individuals who may be experiencing significant depressive symptoms.

### Other variables

Race/ethnicity (Hispanic, Caucasian/White Non-Hispanic, Black/African American, Chinese/Chinese American, Japanese/Japanese American), education attainment (high school or less, some college/technical school, and college or more), physical activity (assessed by modified Kaiser Physical Activity Survey score in housework/caregiving, active living habits, sports, and occupation), health status (excellent, very good, good, fair, and poor), and dietary intake (assessed by food frequency questionnaire) were self-reported during the subject screening and enrollment phase (1995–1997).

At the third follow-up visit, age (years), smoking status (never/former, current smoker), alcohol consumption (alcohol drinker was defined as who had any alcohol in the last 24 h), total family income (< 50,000$, ≥ 50,000$), body mass index (BMI, kg/m^2^), hypertension (the average of three blood pressure readings ≥ 140/90 mmHg or currently taking blood pressure lowering medication), diabetes (self-reported clinical diagnosed of diabetes), sleep medications (not take during the past month, < 1 times per week, 1 to 2 times per week, ≥ 3 times per week), nervous medications, quality of life (score from 0 to 10 score, 0 represents the worst quality and 10 represents the best quality), menopausal status (pre-menopause, early perimenopause, late perimenopause, and natural post menopause), vasomotor symptoms (the frequency of hot flashes or night sweats during the past 2 weeks), estradiol (pg/mL), dehydroepiandrosterone sulfate (ug/dL), follicle-stimulating hormone (mIU/mL), sex hormone-binding globulin (nM), testosterone (ng/dL), total cholesterol (mg/dL), triglycerides (mg/dL), glucose (mg/mL), insulin (uIU/mL), and C-reactive protein (mg/L) were obtained from standardized interviews and measurements. The evaluation and detection methods of the above variables have been described elsewhere^19–24^.

### Statistical analysis

Categorical variables were described by frequency distributions (counts and percentages). After normality testing, all continuous variables were skewed and presented as medians and interquartile ranges (IQR). General characteristics and hematological parameters were summarized and compared using the Chi-square test and the Wilcoxon rank-sum test by the incidence of depressive symptoms. Cox proportional hazard models were fitted to calculate hazard ratios (HRs) and 95% confidence intervals (CIs) for the incidence of depressive symptoms associated with three categories of changes in sleep midpoint (mild-change was used as the reference group). Survival time was defined from the third follow-up visit until the visit of the first depressive symptoms identified or the last follow-up visit. The first model adjusted for age and race/ethnicity. The second model additionally adjusted for education, total family income, smoking status, physical activity score, alcohol consumption, BMI, hypertension, diabetes, health status, and quality of life. The full model additionally adjusted for menopausal status, estradiol, follicle-stimulating hormone, sex hormone-binding globulin, testosterone, and follow-up time. Tests for linear trends were conducted using changes in sleep midpoint as continuous variable in Cox proportional hazard models. Proportional hazards assumptions were verified by assessing Kaplan–Meier survival curves. We performed sensitivity analyses using CES-D score ≥ 20 to identify depressive symptoms. Additionally, we evaluated the impact of additional adjustment for sleep duration and sleep quality. We tested interactions between potential confounding factors and sleep midpoint changes, and further performed subgroup analyses if interactions were statistically significant. We conducted a lag analysis and excluding the follow-up data from the first 4 years. Analyses were executed using Stata version 15.1 (Stata Corporation, College Station, TX, USA). All presented *p*-values are two-sided, and a *P-*value < 0.05 was considered statistically significant.

## Results

### Study sample characteristics

At baseline (the third follow-up visit), the median age was 49 years (IQR 47–51). The proportions of each racial/ethnic women were Caucasian/White Non-Hispanic (48.4%), Black/African American (24.2%), Japanese/Japanese American (12.0%), Chinese/Chinese American (10.2%), and Hispanic (5.2%). 81.6% of women experienced mild sleep midpoint changes, while 6.3% had moderate change and 12.1% had severe changes. The median follow-up time was 7 years (range 1–7 years). Among 1579 women at risk for depressive symptoms at baseline, 496 developed incident depressive symptoms during 8107 person-years of follow-up, an incidence of 61 per 1000 person-years. Compared with women who remained free of depressive symptoms throughout follow-up, those who developed depressive symptoms were more likely to be younger, less educated, less family income, less physically active, lower quality of life, to have worse health status, to have a higher frequency of night sweats, to have worse sleep quality, to have a higher blood glucose, to have a higher total energy intake, to experience a higher frequency of insomnia symptoms, to use sleep medications, and to use nervous medications. Women with incident depressive symptoms had a greater degree of change in the midpoint of sleep compared with those without incident depressive symptoms (Table 1).

**Table 1:** Baseline characteristics of study participants.

	Total participants	Participants without depressive symptoms	Participants with depressive symptoms	P-value
Number of subjects	1579	1083	496	
Follow-up years	7 (1–7)	7 (1–7)	2 (1–7)	< 0.001
Age, years	49 (47–51)	49 (47–51)	48 (46–51)	0.024
Body mass index, kg/m^2^	26.7 (23.0–32.0)	26.6 (23.0–31.8)	26.8 (22.8–32.6)	0.644
Physical activity score	6.7 (5.8–7.5)	6.8 (5.9–7.6)	6.5 (5.7–7.3)	< 0.001
Quality of life	8 (7–9)	8 (8–9)	8 (7–8)	< 0.001
Sleep time, hours	7 (6–7)	7 (6–7)	7 (6–7)	0.198
Dehydroepiandrosterone sulfate, μg/dL	121.5 (81.0–175.1)	119.0 (80.2–175.3)	125.0 (82.3–174.5)	0.529
Follicle-stimulating hormone, mIU/mL	26.1 (13.7–59.3)	27.4 (13.6–61.6)	23.2 (14.0–54.7)	0.176
Sex hormone-binding globulin, nM	39.5 (26.3–54.3)	39.5 (26.3–53.2)	39.4 (26.3–55.5)	0.527
Testosterone, ng/dL	33.5 (24.0–47.8)	33.8 (23.8–47.6)	32.5 (24.3–48.8)	0.700
Estradiol, pg/mL	35.5 (21.1–75.6)	35.1 (20.6–76.8)	36.8 (22.0–71.7)	0.674
Total cholesterol, mg/dL	194 (173–218)	196 (174–219)	193 (171–217)	0.247
Triglycerides, mg/dL	96 (73–140)	94 (72–139)	99 (74–142)	0.255
Glucose, mg/mL	90 (84–97)	89 (84–96)	91 (85–98)	0.014
Insulin, uIU/mL	9.2 (7.1–13.4)	9.1 (7.2–13.2)	9.5 (7.1–13.8)	0.549
C-reactive protein, mg/L	1.6 (0.6–4.6)	1.6 (0.6–4.7)	1.7 (0.6–4.6)	0.819
Total caloric intake, kcal	1701.6 (1339.8–2145.0)	1654.2 (1326.4–2077.2)	1805.7 (1427.4–2317.4)	< 0.001
Caffeine intake, mg	174.7 (63.9–309.1)	173.3 (64.2–307.7)	181.4 (63.1–315.7)	0.722
Race/Ethnicity, n (%)				0.39
Caucasian/White Non-Hispanic	765 (48.4)	534 (49.3)	231 (46.6)	
Black/African American	382 (24.2)	258 (23.8)	124 (25.0)	
Japanese/Japanese American	189 (12.0)	119 (11.0)	70 (14.1)	
Chinese/Chinese American	161 (10.2)	113 (10.4)	48 (9.7)	
Hispanic	82 (5.2)	59 (5.5)	23 (4.6)	
Education, n (%)				0.01
High school or less	311 (19.8)	209 (19.5)	102 (20.7)	
Some college	838 (53.5)	554 (51.6)	284 (57.6)	
College and above	418 (26.7)	311 (28.9)	107 (21.7)	
Total family income, n (%)				< 0.001
< 50,000$	497 (33.1)	308 (29.8)	189 (40.3)	
≥ 50,000$	1004 (66.9)	724 (70.2)	280 (59.7)	
Smoking status, n (%)				0.086
Never	953 (61.4)	673 (63.0)	280 (58.0)	
Former	346 (22.3)	235 (22.0)	111 (23.0)	
Current	252 (16.3)	160 (15.0)	92 (19.0)	
Alcohol consumption, n (%)				0.982
Non-drinker	1190 (84.5)	816 (84.5)	374 (84.4)	
Drinker	219 (15.5)	150 (15.5)	69 (15.6)	
Hypertension, n (%)				0.983
Yes	406 (26.8)	278 (26.8)	128 (26.8)	
No	1109 (73.2)	760 (73.2)	349 (73.2)	
Diabetes, n (%)				0.28
Yes	97 (6.6)	35 (7.6)	62 (6.1)	
No	1382 (93.4)	426 (92.4)	956 (93.9)	
Health status, n (%)				< 0.001
Excellent	386 (24.7)	307 (28.7)	79 (16.2)	
Very good	631 (40.5)	431 (40.2)	200 (50.0)	
Good	402 (25.8)	260 (24.3)	142 (29.1)	
Fair/poor	140 (9.0)	73 (6.8)	67 (13.7)	
Sleep quality, n (%)				< 0.001
Very good	410 (26.0)	326 (30.2)	84 (17.0)	
Fairly good	926 (58.8)	621 (57.4)	305 (61.6)	
Fairly bad	203 (12.9)	117 (10.8)	86 (17.4)	
Very bad	37 (2.3)	17 (1.6)	20 (4%)	
Changes in the midpoint of sleep				0.011
Mild	1289 (81.6)	905 (83.6)	384 (77.4)	
Moderate	99 (6.3)	58 (5.3)	41 (8.3)	
Severe	191 (12.1)	120 (11.1)	71 (14.3)	
Menopausal status, n (%)				0.108
Premenopause	223 (14.1)	168 (15.6)	55 (11.1)	
Early perimenopause	983 (62.3)	659 (60.9)	324 (65.3)	
Late perimenopause	163 (10.4)	114 (10.5)	49 (9.9)	
Natural postmenopause	209 (13.2)	141 (13.0)	68 (13.7)	
Hot flashes during past 2 weeks, n (%)				0.041
≤ 5 Days	1356 (86.1)	942 (87.3)	414 (83.5)	
> 5 Days	219 (13.9)	137 (12.7)	82 (16.5)	
Night sweats during past 2 weeks, n (%)				0.244
≤ 5 Days	1423 (90.5)	983 (91.1)	440 (89.2)	
> 5 Days	149 (9.5)	96 (8.9)	53 (10.8)	
Sleep medication, n (%)				0.014
Not take during the past month	1394 (88.5)	973 (90.0)	421 (85.0)	
< 1 times per week	83 (5.3)	53 (4.9)	30 (6.1%)	
1 to 2 times per week	41 (2.5)	25 (2.3)	16 (3.2)	
≥ 3 times per week	58 (3.7)	30 (2.8)	28 (5.7)	
Insomnia symptoms, n (%)				< 0.001
Yes	133 (8.4)	72 (6.7)	61 (12.3)	
No	1443 (91.6)	1008 (93.3)	435 (87.7)	
Estrogen pills, n (%)				0.674
Yes	22 (1.4)	16 (1.5)	6 (1.2)	
No	1557 (98.6)	1067 (98.5)	490 (98.8)	
Medications for nervous conditions, n (%)				< 0.001
Yes	162 (10.3)	80 (7.4)	82 (16.5)	
No	1417 (89.7)	1003 (92.6)	414 (83.5)	

Data are presented as median (IQR) or n (%).

*P-*value based on χ^2^ test for categorical variables and Wilcoxon rank-sum test for continuous variables.

### Associations between sleep–wake changes and new-onset depressive symptoms

**Figure 1:**
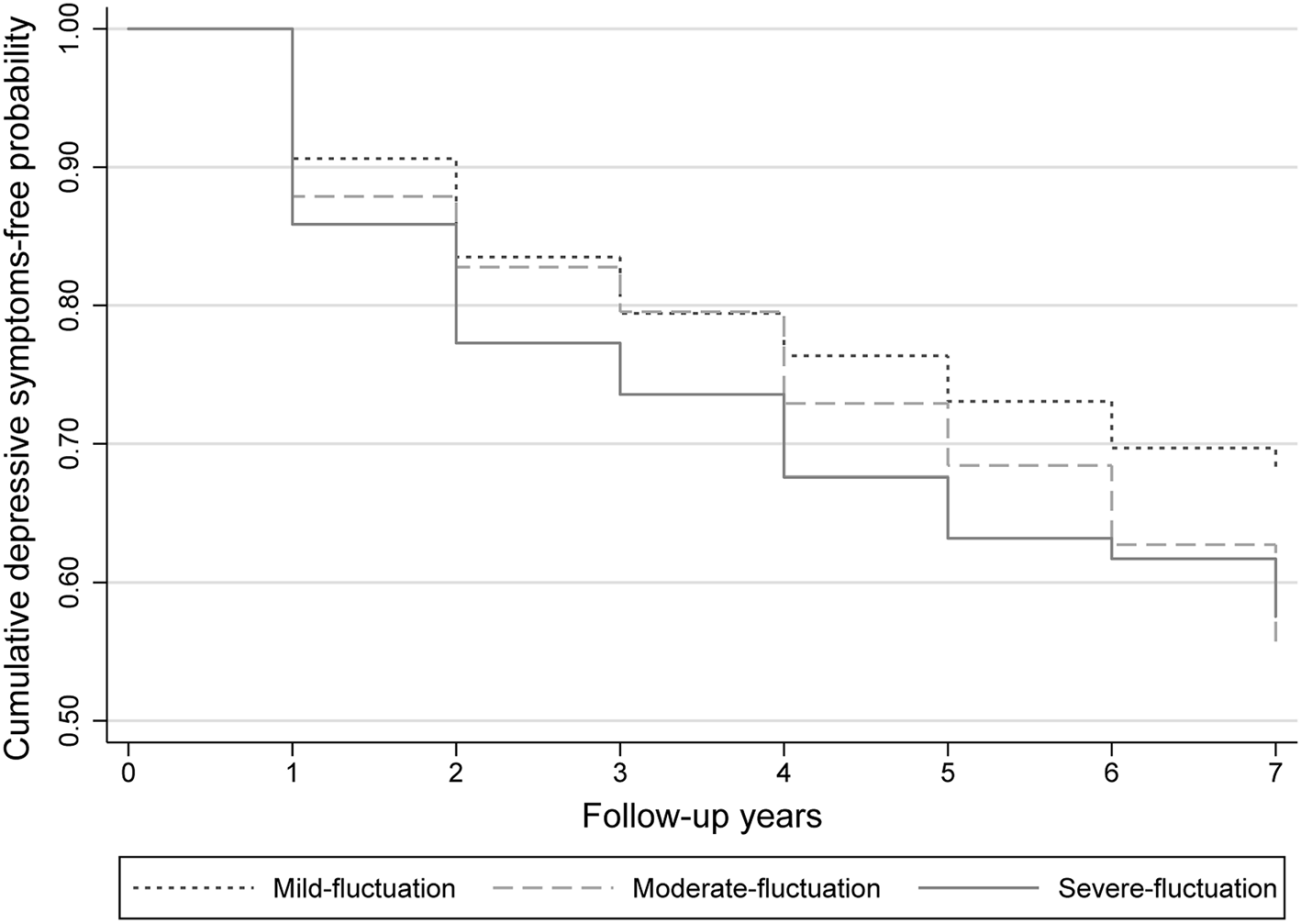
Relationship between changes in the midpoint of sleep and new-onset depressive symptoms.

The associations between changes in the midpoint of sleep and new-onset depressive symptoms are presented in Fig. 1 and Table 2. After adjusting for age and race/ethnicity (model 1), women with greater sleep midpoint changes had significantly higher risk for depressive symptoms (*p*-value for trend = 0.003). After further adjusting for lifestyle and health-related covariates (model 2), the HR (95% CI) of depressive symptoms for severe sleep midpoint change was 1.51 (1.12, 2.05) compared with mild sleep midpoint change. This relationship remained statistically significant and changed little when additionally controlling for menopausal status and sex hormones (model 3; HR 1.57, 95% CI 1.16, 2.14, *P*-value for trend = 0.002).

**Table 2:** Associations between sleep midpoint changes and risk of depressive symptoms.

	Mild	Moderate	*P*-value	Severe	*P*-value	*P*-value for trend
	HR (95%CI)	HR (95% CI)		HR (95% CI)		
No. cases/person-years	1289/6759	99/510		191/838		
Model 1	1.00 (reference)	1.40 (1.01, 1.93)	0.044	1.42 (1.09, 1.86)	0.009	0.003
Model 2	1.00 (reference)	1.44 (0.98, 2.12)	0.064	1.51 (1.12, 2.05)	0.008	0.003
Model 3	1.00 (reference)	1.42 (0.96, 2.11)	0.075	1.57 (1.15, 2.14)	0.004	0.002

Model 1: Age and Race/Ethnicity.

Model 2: Model 1 plus education, family income, body mass index, smoking status, alcohol consumption, physical activity score, hypertension status, diabetes status, health status, and quality of life.

Model 3: Model 2 plus menopausal status, dehydroepiandrosterone sulfate, follicle-stimulating hormone, sex hormone-binding globulin, testosterone, estradiol, and follow-up period.

### Sensitivity analyses

We explored the potential effects of depressive cut-off point, caffeine intake, sleep duration, sleep quality, insomnia symptoms, use of sleep medications, and use of nervous medications in the model, and findings were consistent with the main models (Supplementary Tables 1, 2). There were no significant interactions between sleep midpoint changes and covariates (Supplementary Table 3). After conducting a lag analysis and excluding the follow-up data from the first four years, we found that the correlation still remains statistically significant and even stronger (HR 2.04, 95% CI 1.09, 3.82, *P*-value for trend = 0.008). After excluding women who identified as shiftworkers (n = 54), the relationship still remains statistically significant (HR 1.64, 95% CI 1.20, 2.45, *P*-value for trend = 0.001).

## Discussion

This large-sample prospective cohort study supports the hypothesis that greater sleep–wake changes are associated with higher risk for depressive symptoms in women during the menopausal transition. In the present study, 81.6% of women experienced mild sleep midpoint changes (sleep midpoint changes < 1 h), 6.3% of having moderate sleep–wake changes (sleep midpoint changes between 1 to 2 h) and 12.1% having severe changes (sleep midpoint changes > 2 h). Findings indicates that women with severe sleep midpoint change have a 57% higher risk of depressive symptoms compared with women with mild sleep midpoint change. This relationship is independent of traditional depression risk factors, menopausal status, and sex hormones levels.

Previous studies on the association between sleep midpoint and depressive symptoms examined sleep midpoint at only one time point, which failed to capture long-term changes in individual sleep–wake rhythms^13,25–27^. Not surprisingly, variability of sleep midpoint between individuals can be considerable, particularly in the absence of social constraints^28^. Twin and family studies suggest that genetic factors account for a substantial proportion (up to 50%) of population variability in circadian timing^29^. To minimize the effect of inter-individual variation, we assess changes in sleep–wake rhythm by calculating the difference in sleep midpoint between two consecutive years for each participant. Thus, the current study is the first to describe changes in the midpoint of sleep and to track the incidence of subsequent depressive symptoms among midlife women.

We seek to explore the underlying mechanisms of the interaction between sleep–wake changes and depressive symptoms. Previous studies have shown that circadian rhythm disruption is prone to metabolic abnormalities (mainly manifested as glucose, lipid, and energy metabolism), which in turn may increase the risk of depressive symptoms^30–32^. In our study, after adjusting for indicators of metabolic abnormalities (energy intake, insulin, glucose, and lipids; Supplementary Table 2, model 5) and metabolic diseases (hypertension and diabetes), a high risk of depressive symptoms is also observed in patients with severe sleep midpoint change. Thus, metabolic alterations do not appear to explain the association between sleep–wake changes and depression symptoms. Furthermore, we considered interactions between the circadian system and the sleep homeostatic system, the latter is the main driving force for maintaining sleep duration and intensity. Several studies have shown that sleep duration and quality are independently associated with incident depressive symptoms^2,33^. In our study, the association between sleep–wake changes and depressive symptoms persisted after adjusting for sleep duration and sleep quality. Thus, the influence of the sleep–wake changes on depressive symptoms may be independent of the sleep homeostasis system. During the menopausal transition, with estrogen levels decline, women often experience vasomotor symptoms, which are associated with both sleeping problems and depressive symptoms^34^; however, associations between sleep–wake changes and depressive symptoms consistent with the primary models when further adjusting for vasomotor symptoms (Supplementary Table 2, model 6). Moreover, we consider the role of inflammation, the associations persist after adjusting C-reactive protein (Supplementary Table 2, model 7). Other potential mechanisms, such as melatonin, cortisol, and epigenetics, remain to be explored in depth^35,36^.

Our study has several limitations. Sleep data used in this study are obtained by self-report, which is less objective than polysomnography. Although subjective measures have their limitations, study has shown that there is a moderate correlation between subjective sleep assessments and objective measurements, such as polysomnography^37^. Moreover, subjective measurement methods are easier to implement and do not require expensive equipment or the involvement of specialized technicians, which allows the study to be conducted on a broader sample, thereby enhancing the practical application value of the research. Our study is limited to women during the menopausal transition, so whether the findings can be extended to other age groups of women or men still needs further investigation. Although we discuss a wide range of covariates, the possibility that the observed associations are disturbed by unmeasured or residual confounding factors cannot be ruled out. Unfortunately, only two consecutive sleep midpoint data are available for analysis, making it impossible to explore the associations between longer-term sleep–wake change signatures and depressive symptoms. The sleep midpoint is only an indirect measure of the circadian rhythm and cannot fully represent an individual’s sleep–wake pattern or physiological rhythm.

## Conclusions

In conclusion, this prospective cohort study of middle-aged women supports the hypothesis that larger sleep–wake changes are associated with higher risk for depressive symptoms, after considering metabolic abnormalities, sleep homeostasis system, sex hormones, and vasomotor symptoms. These findings highlight the importance of considering sleep–wake changes when performing a comprehensive sleep assessment and underscore that chronic exposure to severe sleep–wake change increases the risk of depressive symptoms by at least 50% in women during the menopausal transition.

### Supplementary Information

Supplementary Tables.

## Data Availability

Data are publicly available at https://www.swanstudy.org/swan-research/data-access.
