# Supplementary material for: Sleep–wake changes and incident depressive symptoms in midlife women
Source: Sci Rep. 2024 Jul 2;14:15184. doi: 10.1038/s41598-024-66145-3 (PMC11219764; doi:10.1038/s41598-024-66145-3)
Supplement: Supplementary file 1 — Supplementary Tables. [file 41598_2024_66145_MOESM1_ESM.docx]

**Supplementary table 1.** Associations between sleep midpoint fluctuations and risk of depressive symptoms (CES-D score ≥ 20)

|  | **Mild** | **Moderate** |  | **Severe** |  |  |
| --- | --- | --- | --- | --- | --- | --- |
|  | **HR (95%CI)** | **HR (95%CI)** | ***P*-**  **value** | **HR (95%CI)** | ***P*-**  **value** | ***P*-value**  **for trend** |
| **No. cases/person-years** | 1289/8121 | 99/610 |  | 191/1159 |  |  |
| **Model 1** | 1.00 (reference) | 1.41 (0.93, 2.13) | 0.106 | 1.42 (1.01, 1.99) | 0.043 | 0.021 |
| **Model 2** | 1.00 (reference) | 1.47 (0.90, 2.39) | 0.122 | 1.53 (1.04, 2.25) | 0.031 | 0.017 |
| **Model 3** | 1.00 (reference) | 1.44 (0.89, 2.36) | 0.141 | 1.59 (1.08, 2.36) | 0.019 | 0.011 |

Model 1: Age and Race/Ethnicity;

Model 2: Model 1 plus education, family income, body mass index, smoking status, alcohol consumption, physical activity score, hypertension status, diabetes status, health status, quality of life;

Model 3: Model 2 plus menopausal status, dehydroepiandrosterone sulfate, follicle-stimulating hormone, sex hormone-binding globulin, testosterone, estradiol.

**Supplementary table 2.** Associations between sleep midpoint fluctuations and risk of depressive symptoms after further adjusting for variables related to sleep homeostasis, metabolism, and vasomotor symptoms

|  | **Mild** | **Moderate** |  | **Severe** |  |  |
| --- | --- | --- | --- | --- | --- | --- |
|  | **HR (95%CI)** | **HR (95%CI)** | ***P*-**  **value** | **HR (95%CI)** | ***P*-**  **value** | ***P*-value**  **for trend** |
| No. cases/person-years | 1289/8121 | 99/610 |  | 191/1159 |  |  |
| Model 4 | 1.00 (reference) | 1.60 (1.08, 2.37) | 0.020 | 1.60 (1.16, 2.21) | 0.004 | 0.001 |
| Model 5 | 1.00 (reference) | 1.52 (1.02, 2.28) | 0.041 | 1.57 (1.13, 2.18) | 0.007 | 0.003 |
| Model 6 | 1.00 (reference) | 1.51 (1.01, 2.27) | 0.045 | 1.58 (1.14, 2.20) | 0.006 | 0.002 |
| Model 7 | 1.00 (reference) | 1.54 (1.02, 2.31) | 0.038 | 1.62 (1.15, 2.27) | 0.005 | 0.002 |

Model 4: Model 3 plus caffeine intake, sleep duration, sleep quality, insomnia symptoms, use of sleep medications, and use of nervous medications;

Model 5: Model 4 plus total cholesterol, triglycerides, glucose, insulin, and total caloric intake;

Model 6: Model 5 plus hot flashes and night sweats;

Model 7: Model 6 plus C-reactive protein.

**Supplementary table 3.** Interactions between sleep midpoint fluctuations and covariates

|  | **Mild** | **Moderate** |  | **Severe** |  |
| --- | --- | --- | --- | --- | --- |
|  | **HR (95%CI)** | **HR (95%CI)** | ***P*-value for interaction** | **HR (95%CI)** | ***P*-value for interaction** |
| **Age** | 1.00 (reference) | 1.04 (0.91, 1.17) | 0.582 | 1.03 (0.94, 1.13) | 0.476 |
| **Body mass index** | 1.00 (reference) | 1.00 (0.96, 1.04) | 0.919 | 1.02 (0.99, 1.05) | 0.197 |
| **Physical activity score** | 1.00 (reference) | 1.02 (0.77, 1.34) | 0.901 | 1.01 (0.84, 1.22) | 0.907 |
| **Quality of life** | 1.00 (reference) | 1.05 (0.84, 1.32) | 0.651 | 1.06 (0.91, 1.24) | 0.442 |
| **Sleep time, hours** | 1.00 (reference) | 1.03 (0.80, 1.34) | 0.790 | 1.09 (0.91, 1.31) | 0.354 |
| **Dehydroepiandrosterone sulfate** | 1.00 (reference) | 1.003 (0.999, 1.007) | 0.175 | 1.000 (0.997, 1.004) | 0.790 |
| **Follicle-stimulating hormone** | 1.00 (reference) | 0.997 (0.989, 1.005) | 0.474 | 0.999 (0.993, 1.006) | 0.942 |
| **Sex hormone-binding globulin** | 1.00 (reference) | 0.999 (0.984, 1.013) | 0.903 | 0.999 (0.988, 1.012) | 0.990 |
| **Testosterone** | 1.00 (reference) | 1.007 (0.992, 1.022) | 0.363 | 1.001 (0.987, 1.015) | 0.896 |
| **Estradiol** | 1.00 (reference) | 1.003 (0.998, 1.008) | 0.218 | 0.999 (0.994, 1.004) | 0.667 |
| **Race/Ethnicity** |  |  |  |  |  |
| Black/African American | 1.00 (reference) |  |  |  |  |
| Chinese/Chinese American | 1.00 (reference) | 1.05 (0.23, 4.90) | 0.945 | 0.95 (0.31, 2.87) | 0.923 |
| Japanese/Japanese American | 1.00 (reference) | 1.16 (0.36, 3.73) | 0.801 | 0.47 (0.11, 2.06) | 0.321 |
| Caucasian/White Non-Hispanic | 1.00 (reference) | 1.51 (0.71, 3.21) | 0.280 | 1.43 (0.79, 2.59) | 0.231 |
| Hispanic | 1.00 (reference) | 1.32 (0.68, 2.60) | 0.405 | 0.99 (0.37, 2.61) | 0.984 |
| **Education** |  |  |  |  |  |
| High school or less | 1.00 (reference) |  |  |  |  |
| Some college | 1.00 (reference) | 0.77 (0.37, 1.62) | 0.497 | 0.93 (0.52, 1.66) | 0.799 |
| College and above | 1.00 (reference) | 0.72 (0.24, 2.11) | 0.547 | 0.96 (0.44, 2.11) | 0.93 |
| **Total family income** |  |  |  |  |  |
| < 50,000$ | 1.00 (reference) |  |  |  |  |
| ≥ 50,000$ | 1.00 (reference) | 1.37 (0.71, 2.65) | 0.345 | 1.05 (0.62, 1.79) | 0.844 |
| **Smoking status** |  |  |  |  |  |
| Never | 1.00 (reference) |  |  |  |  |
| Former | 1.00 (reference) | 1.35 (0.58, 3.11) | 0.486 | 1.16 (0.52, 2.57) | 0.715 |
| Current | 1.00 (reference) | 0.73 (0.36, 1.45) | 0.365 | 0.97 (0.52, 1.80) | 0.928 |
| **Alcohol consumption** |  |  |  |  |  |
| Non-drinker | 1.00 (reference) |  |  |  |  |
| Drinker | 1.00 (reference) | 0.62 (0.14, 2.66) | 0.522 | 1.06 (0.46, 2.44) | 0.886 |
| **Hypertension** |  |  |  |  |  |
| No | 1.00 (reference) |  |  |  |  |
| Yes | 1.00 (reference) | 0.89 (0.42, 1.91) | 0.770 | 1.48 (0.87, 2.52) | 0.149 |
| **Diabetes** |  |  |  |  |  |
| No | 1.00 (reference) |  |  |  |  |
| Yes | 1.00 (reference) | 0.53 (0.07, 3.98) | 0.534 | 0.88 (0.35, 2.20) | 0.779 |
| **Health status** |  |  |  |  |  |
| Excellent | 1.00 (reference) |  |  |  |  |
| Very good | 1.00 (reference) | 0.74 (0.29, 1.90) | 0.530 | 0.80 (0.37, 1.73) | 0.570 |
| Good | 1.00 (reference) | 0.57 (0.22, 1.47) | 0.244 | 0.49 (0.21, 1.11) | 0.087 |
| Fair/poor | 1.00 (reference) | 0.27 (0.06, 1.08) | 0.064 | 0.46 (0.18, 1.17) | 0.105 |
| **Sleep quality** |  |  |  |  |  |
| Very good | 1.00 (reference) |  |  |  |  |
| Fairly good | 1.00 (reference) | 0.76 (0.34, 1.72) | 0.517 | 0.80 (0.40, 1.60) | 0.526 |
| Fairly bad | 1.00 (reference) | 0.81 (0.27, 2.41) | 0.710 | 0.89 (0.37, 2.12) | 0.787 |
| Very bad | 1.00 (reference) | 0.49 (0.09, 2.53) | 0.397 | 0.66 (0.19, 2.36) | 0.526 |
| **Menopausal status** |  |  |  |  |  |
| Natural postmenopause | 1.00 (reference) |  |  |  |  |
| Late perimenopause | 1.00 (reference) | 0.86 (0.18, 4.08) | 0.853 | 0.38 (0.13, 1.15) | 0.087 |
| Early perimenopause | 1.00 (reference) | 1.32 (0.39, 4.50) | 0.652 | 0.87 (0.45, 1.68) | 0.675 |
| Premenopause | 1.00 (reference) | 0.70 (0.11, 4.37) | 0.702 | 0.59 (0.29, 1.75) | 0.342 |

|  |
| --- |
